# Involvement of TRPA1 activation in acute pain induced by cadmium in mice

**DOI:** 10.1186/1744-8069-9-7

**Published:** 2013-02-28

**Authors:** Saeko Miura, Kenji Takahashi, Toshiaki Imagawa, Kunitoshi Uchida, Shigeru Saito, Makoto Tominaga, Toshio Ohta

**Affiliations:** 1 Department of Veterinary Pharmacology, Faculty of Agriculture, Tottori University, Tottori 680-8553, Japan; 2 Biological Chemistry, Department of Chemistry, Faculty of Science, Hokkaido University, Sapporo 060-0810, Japan; 3 Division of Cell Signaling, Okazaki Institute for Integrative Bioscience (National Institute for Physiological Sciences), National Institutes of Natural Sciences, Okazaki 444-8787, Japan

**Keywords:** Cadmium, Intracellular calcium, Mouse, Pain, Patch-clamp, Sensory neuron, TRPA1

## Abstract

**Background:**

Cadmium (Cd) is an environmental pollutant and acute exposure to it causes symptoms related to pain and inflammation in the airway and gastrointestinal tract, but the underlying mechanisms are still unclear. TRPA1 is a nonselective cation channel expressed in sensory neurons and acts as a nociceptive receptor. Some metal ions such as Ca, Mg, Ba and Zn are reported to modulate TRPA1 channel activity. In the present study, we investigated the effect of Cd on cultured mouse dorsal root ganglion neurons and a heterologous expression system to analyze the effect of Cd at the molecular level. In addition, we examined whether Cd caused acute pain *in vivo*.

**Results:**

In wild-type mouse sensory neurons, Cd evoked an elevation of the intracellular Ca concentration ([Ca^2+^]_i_) that was inhibited by external Ca removal and TRPA1 blockers. Most of the Cd-sensitive neurons were also sensitive to cinnamaldehyde (a TRPA1 agonist) and [Ca^2+^]_i_ responses to Cd were absent in TRPA1(−/−) mouse neurons. Heterologous expression of TRPA1 mutant channels that were less sensitive to Zn showed attenuation of Cd sensitivity. Intracellular Cd imaging revealed that Cd entered sensory neurons through TRPA1. The stimulatory effects of Cd were confirmed in TRPA1-expressing rat pancreatic cancer cells (RIN-14B). Intraplantar injection of Cd induced pain-related behaviors that were largely attenuated in TRPA1(−/−) mice.

**Conclusions:**

Cd excites sensory neurons via activation of TRPA1 and causes acute pain, the mechanism of which may be similar to that of Zn. The present results indicate that TRPA1 is involved in the nociceptive or inflammatory effects of Cd.

## Background

Cadmium (Cd) is a ubiquitous environmental pollutant distributed in rocks, soil, the atmosphere and water [1]. Human exposure occurs mainly from consumption of contaminated food, smoking and inhalation by workers in metal industries (reviewed by [2,3]). Cd is more efficiently absorbed by the lungs (25-50%) than the gastrointestinal tract (5%) [4]. Thus, Cd inhalation is often a problem in metal workers and cigarette smokers.

Chronic Cd toxicity has been well studied and the main target organs are the kidney, liver, lung cardiovascular, immune and reproductive systems (reviewed by [5]), with resultant, renal tubular dysfunction and subsequent induction of osteomalacia known as “itai-itai disease” (reviewed by [6]). In acute exposure, on the other hand, Cd causes symptoms related to pain and inflammation in the airway or gastrointestinal tract. After exposure to a high level of metal dust or fumes containing Cd causes irritation of the upper respiratory tract and induces coughing in the early stage, various clinical manifestations known as metal fume fever occur, including airway inflammation, pulmonary edema, chest pain and flu-like symptoms. Cd ingestion causes abdominal pain, severe nausea and diarrhea (reviewed by [2]). These reports suggest that Cd stimulates sensory processing involved in pain and inflammation, but the underlying mechanisms are unclear.

TRPA1 and TRPV1 are nonselective cation channels expressed in nociceptive neurons and act as polymodal receptor [7]. For example, TRPA1 channel is activated by a range of natural products (allyl isothiocyanate, cinnamaldehyde and allicin found in mustard oil, cinnamon, and garlic, respectively) [8,9], environmental irritants (acrolein and formalin) [10,11], α,β-unsaturated aldehyde (cigarette smoke) [12], reactive oxygen species [13] and cold temperature [14], and contributes to the perception of noxious stimuli. TRPV1 is also activated by various stimuli such as capsaicin, protons, and noxious heat, and plays an important role in sensory transduction [15,16].

It has been reported that some metal ions regulate TRP channel activities. For example, nickel activates TRPV1 in the mM range, which is associated with nickel-induced contact dermatitis [17]. It has also been reported that Na, Mg and Ca are able to activate TRPV1, which contributes to the nociceptive responses to elevated ionic strength [18]. Moreover, Na negatively regulates TRPV1, since removal of external Na activates TRPV1 [19].

Some metal ions such as Ca, Mg, Ba and Zn modulate TRPA1 channel activity [20-23]. Among them, Zn, in the same metal ionic group as Cd, stimulates sensory nerves and causes nociception in mice through direct activation of TRPA1 [21,22].

In the present study, we examined the effect of Cd on sensory neurons *in vitro* and on nociceptive behavior *in vivo* using wild-type and TRPA1(−/−) mice. To examine the neuronal activity, we used fura-2-based Ca-imaging techniques since some TRP channels are highly Ca permeable [24]. We investigated the effect of Cd on cultured mouse dorsal root ganglion (DRG) neurons, which are a useful model of nociception *in vitro*. We also used a heterologous expression system to analyze the effect of Cd at the molecular level using Ca-imaging and patch-clamp techniques. In addition, we examined whether Cd induced acute pain *in vivo*.

## Results

### Cd-induced [Ca^2+^]_i_ increase in mouse DRG neurons

**Figure 1:**
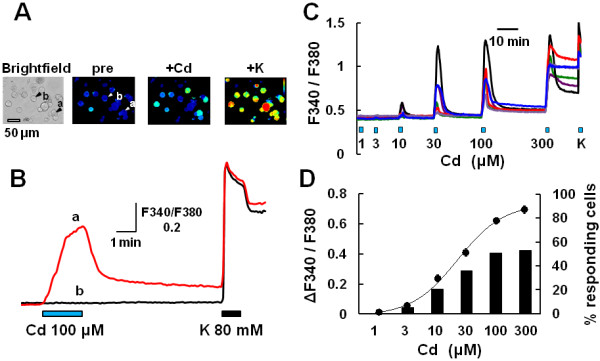
**[Ca^2+^]_i_ increases induced by Cd in a subset of mouse sensory neurons. **(**A**) Cd evokes a [Ca^2+^]_i_ increase in cultured mouse DRG neurons loaded with fura-2. An image under transmitted light (left), and pseudocolor images (right), before (pre) and after the application of 100 μM Cd (+Cd), and 80 mM KCl (+K). In a bright field image, cells with an arrow (a, b) correspond to (**B**). (**B**) Actual recordings of changes in [Ca^2+^]_i_ responses to Cd and KCl in KCl-responding cells (neurons). (**C**) Actual recordings of the changes in [Ca^2+^]_i_ induced by increasing concentrations of Cd (1–300 μM). (**D**) Circles and columns show the concentration-response curve for Cd-induced [Ca^2+^]_i_ increases and the percentage of Cd-responding neurons among all neurons, respectively. The percentages of Cd-responding cells were calculated from the percentage obtained with each coverslip. Symbols with vertical lines show mean ± SEM (n=131, from 3 mice).

Using the Ca sensitive dye fura-2, we examined the effect of Cd on changes in the fura-2 ratio, which reflects the intracellular Ca concentration ([Ca^2+^]_i_). Cells were stimulated with Cd (100 μM) for 2 min and subsequently with KCl (80 mM) for 1 min. Actual traces of [Ca^2+^]_i_ showed that Cd elicited a [Ca^2+^]_i_ increase in some cells responding to KCl (i.e., neurons) (Figure 1A and B). [Ca^2+^]_i_ was increased by increasing concentrations of Cd (1–300 μM). [Ca^2+^]_i_ responses to Cd peaked during the application to Cd, then returned to the basal level (<100 μM), but were sustained even after its washout at 300 μM (Figure 1C). Figure 1D shows the concentration-response relations for Cd; the EC_50_ value was 21.9±3.4 μM. The percentages of Cd-responding neurons increased with increasing concentrations of Cd.

### Involvement of TRPA1 in the Cd-induced [Ca^2+^]_i_ increase

**Figure 2:**
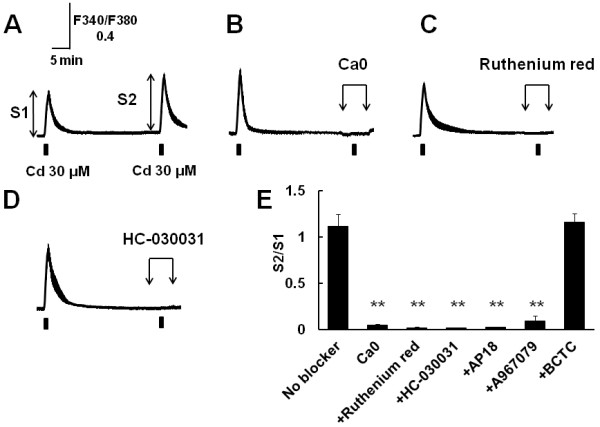
**Inhibition of Cd-induced [Ca^2+^]_i_ increase by removal of extracellular Ca and TRPA1 blockers. **(**A**) [Ca^2+^]_i_ responses in mouse DRG neurons induced by repetitive stimuli with Cd (30 μM, 1 min) with an interval of 30 min. (**B**) Removal of extracellular Ca, (**C**) application of ruthenium red (10 μM) or (**D**) HC-030031(10 μM) was performed before 2 min, during and after 2 min application of Cd. Traces shows mean ± SEM of representative data from 8–17 cells. (**E**) Summarized effects of these pharmacological treatments. S1 and S2 are the first peak amplitude and the second one without (No blocker) and with these treatments, respectively. Data are shown as S2/S1. (No blocker; n=13, Ca0; n=29, ruthenium red; n=39, HC-030031; n=16, AP18 (10 μM); n=22, A967079 (10 μM); n=18, BCTC (10 μM); n=32, from 3 mice) **,P<0.01 vs. No blocker.

Next, we examined the effects of removal of extracellular Ca and TRP channel blockers. Repetitive application of Cd (30 μM, 1 min) after an interval of 30 min induced similar [Ca^2+^]_i_ increases (Figure 2A). Then the second application was carried out in the absence of extracellular Ca (Figure 2B) or presence of TRP channel blockers (Figure 2C, D). Removal of extracellular Ca abolished the Cd-induced [Ca^2+^]_i_ increase, indicating that Cd-induced [Ca^2+^]_i_ elevation resulted from extracellular Ca influx. Ruthenium red (10 μM), a broad TRP channel blocker, and HC-030031 (10 μM), AP18 (10 μM) and A967079 (10 μM), specific TRPA1 blockers, but not BCTC (10 μM), a TRPV1 blocker, inhibited the Cd-induced [Ca^2+^]_i_ increase (Figure 2E). These pharmacological analyses suggested that Cd evoked the [Ca^2+^]_i_ increase in mouse sensory neurons through the activation of TRPA1.

### Absence of [Ca^2+^]_i_ response to Cd in TRPA1(−/−) mouse DRG neurons

**Figure 3:**
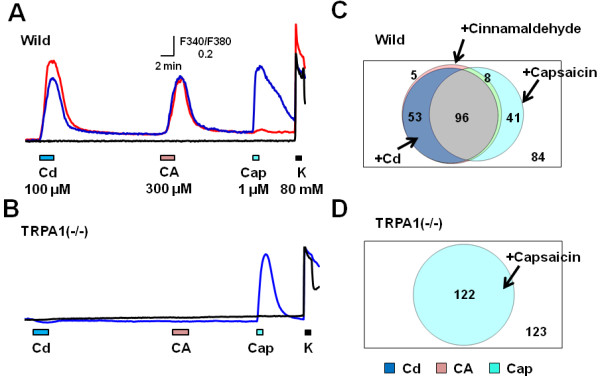
**Cd-induced [Ca^2+^]_i_ increase is absent in TRPA1 (−/−) mouse DRG neurons.** Actual recordings of [Ca^2+^]_i_ responses to sequential application of Cd (100 μM, 2 min), cinnamaldehyde (CA, 300 μM, 2 min), capsaicin (Cap, 1 μM, 1 min), and KCl (K, 80 mM, 1 min) in wild-type (**A**) and TRPA1(−/−) (**B**) mouse DRG neurons. Venn diagram showing the sensitivities to Cd, cinnamaldehyde, capsaicin and KCl in wild-type (**C**; n=299 from 5 mice) and in TRPA1(−/−) DRG neurons (**D**; n=245 from 4 mice). Numerics indicate the number of cells responding to each stimulus. Those in the outermost frames express the number of neurons responding to KCl alone. Note that Cd-responding neurons are mostly coincident with CA-responding ones in the wild-type but they were absent in TRPA1(−/−) mouse DRG neurons.

To verify the relationship between Cd and TRPA1, we used TRPA1(−/−) mice. Figure 3A and B show actual traces of [Ca^2+^]_i_ responses to Cd (100 μM) and subsequent cinnamaldehyde (CA, a TRPA1 agonist, 300 μM), capsaicin (a TRPV1 agonist, 1 μM) and KCl (80 mM) in wild-type and TRPA1(−/−) mouse DRG neurons, respectively. In wild-type mouse DRG neurons, most of the Cd-sensitive cells were also CA sensitive (Figure 3C). In the TRPA1(−/−) mouse, on the other hand, Cd failed to increase [Ca^2+^]_i_ (Figure 3B, D). These data clearly indicated that TRPA1 was involved in the Cd-induced [Ca^2+^]_i_ increases in mouse sensory neurons.

### Cd-influx through TRPA1

**Figure 4:**
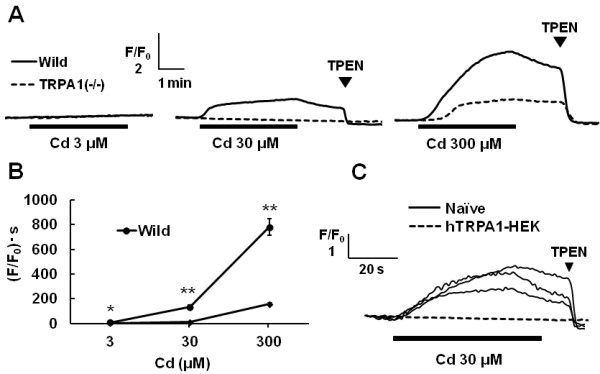
**Cd-influx into the cells through TRPA1. **(**A**) Actual recordings of the changes in the fluorescent ratio induced by Cd in Leadmium Green-loaded wild-type and TRPA1(−/−) mouse DRG neurons. Cells were exposed to Cd (3–300 μM, 4 min) and then TPEN (0.05 mM, 1 min), a cell-permeable heavy metal chelator, was applied at the points shown by arrowheads. Solid and broken lines show the responses in wild-type and TRPA1(−/−) mouse DRG neurons, respectively. (**B**) The area under the curve of the fluorescent change after the administration of Cd to wild-type and TRPA1(−/−) mouse DRG neurons. Symbols with vertical lines show mean ± SEM. (wild-type; n=112-243, TRPA1(−/−); n=193-382, from 3 mice). *,*P* <0.05, **,*P* <0.01 vs. TRPA1(−/−). (**C**) Solid and broken lines show actual recordings of the changes in the fluorescent ratio induced by Cd in human TRPA1-expresing HEK293 cells (hTRPA1-HEK) and untransfected HEK293 cells (Naïve), respectively.

As shown in Figure 1C, when Cd was applied at the concentration of 300 μM, a sustained fura-2 ratio rise was observed even after the washout of Cd. This may have been due to Cd entry into the cell, since fura-2 is sensitive to not only Ca, but also Cd [25]. Thus, we used Leadmium Green, a Cd indicator for intracellular Cd imaging to examine the possibility of Cd influx. Cd induced an increase of the fluorescent intensity of Leadmium Green-loaded wild-type mouse DRG neurons, whereas its intensity was significantly less in TRPA1(−/−) mouse DRG neurons (Figure 4), suggesting that Cd entered DRG neurons through TRPA1 channels. To confirm Cd influx into cells through TRPA1 channel, we carried out Cd-imaging in human TRPA1-expressing HEK293 cells. As shown in Figure 4C, Cd evoked increases of the fluorescence of Leadmium Green in HEK293 cells expressing human TRPA1 but not in untransfected HEK293 cells.

### [Ca^2+^]_i_ responses to Cd in TRPA1-expressing RIN-14B rat pancreatic cancer cells

**Figure 5:**
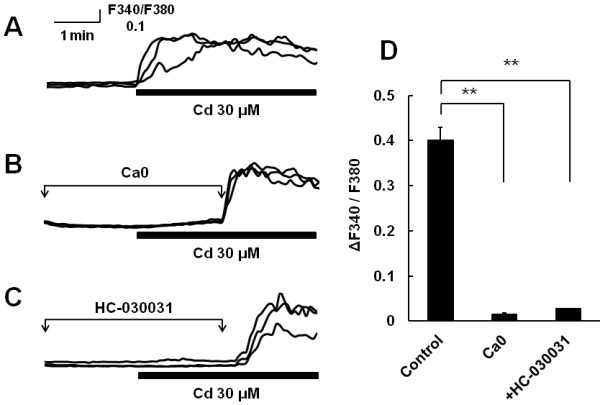
**Cd-induced [Ca^2+^]_i_ increases in RIN-14B rat pancreatic cancer cells. **(**A**) Actual traces of [Ca^2+^]_i_ responses to Cd (30 μM, 4 min) in RIN-14B cells. (**B**) Effect of removal of extracellular Ca (Ca0) and (**C**) HC-030031 (10 μM) on the Cd-induced [Ca^2+^]_i_ increases. Extracellular Ca was removed or a TRPA1 blocker was applied before and during 2 min application of Cd. (**D**) Summarized effects of these treatments. Columns show the increments of [Ca^2+^]_i_ in response to Cd. Symbols with vertical lines show mean ± SEM. (Control; n=33, Ca0; n=72, HC-030031; n=34, from 2 experiments) **,P <0.01.

To confirm the effect of Cd on TRPA1, we used RIN-14B cells, rat enterochromaffin cell line, which express TRPA1 endogenously [26]. As shown in Figure 5, Cd (30 μM, 4 min) induced [Ca^2+^]_i_ increases in RIN-14B cells that were suppressed by removal of extracellular Ca or the application of HC-030031 (10 μM).

### Effect of Cd on TRPA1 mutant channels less sensitive to Zn

**Figure 6:**
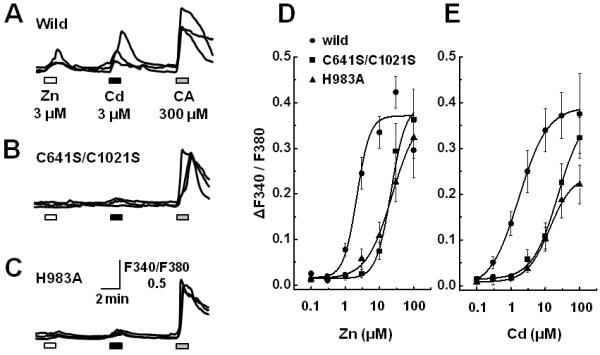
**Involvement of three amino acid residues of TRPA1 in activation by Cd.** Actual traces of [Ca^2+^]_i_ responses to Zn (3 μM, 2 min), Cd (3 μM, 2 min) and cinnnamaldehyde (CA, 300 μM) in HEK293 cells expressing wild-type (**A**), C641S/C1021S (**B**) and H983A TRPA1 (**C**). Concentration-response curves for Zn (**D**) and Cd (**E**) in wild-type and the two types of mutant TRPA1-expressing cells. (wild-type; n=10-40 cells, from 2 experiments, C641S/C1021S; n=13-36, from 2–3 experiments, H983A; n=17-50, from 2–4 experiments).

Zn, in the same metal ionic group as Cd, has been shown to be an agonist of TRPA1 [21,22]. Thus, we hypothesized that Cd recognized the same amino acid residues to activate TRPA1 as Zn. We used two mutant TRPA1 channels in which three amino acids were replaced by others (C641S/C1021S, H983A). As shown in Figure 6, these mutant channels exhibited low responsiveness to Zn (3 μM). (EC_50_ values for Zn: 2.2±0.7 μM for wild-type, 21.6±7.8 μM for C641S/C1021S, 23.4±8.2 for H983A). Similarly, these mutant channels showed significant attenuation of the [Ca^2+^]_i_ responses to Cd (3 μM) and the EC_50_ values were about 10-fold higher than for wild-type TRPA1 channels, though C641S/C1021S mutant TRPA1 did not reached to the maximum (1.7±0.08 μM for wild-type, 23.4±8.2 μM for C641S/C1021S, 13.4±2.0 μM for H983A).

**Figure 7:**
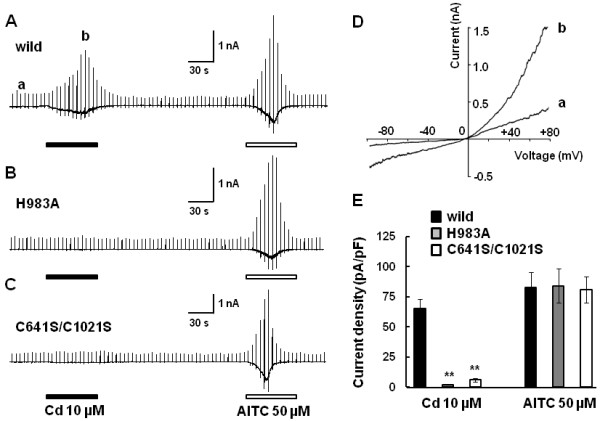
**Cd activates wild-type human TRPA1 but less mutant channels.** Representative traces of the whole-cell currents activated by Cd (10 μM) followed by ally isothiocyanate (AITC, 50 μM) in HEK293 cells expressing wild type (**A**), H983A (**B**) and C641S/C1021S (**C**) TRPA1. (**D**) A current–voltage (I-V) curve of the Cd-activated current showing an outward rectification. Alphabet small letters (a, b) correspond to those at the time points in A. (**E**) Quantification of the current densities activated by Cd (10 μM) and AITC (50 μM) in HEK293 cells expressing human TRPA1; wild, a H983A mutant and C641S/C1021S mutant. Ordinates show inward current density (pA/pF) at −60 mV. Each column represents the mean with SEM. (wild-type; n=8, H983A; n=6, C641S/C1021S; n=6) **, *P*<0.01 vs. wild.

To obtain direct evidence for Cd-induced TRPA1 channel activation, we performed whole-cell patch-clamp experiments in HEK293 cells expressing hTRPA1. Figure 7A shows a representative whole-cell current evoked by 10 μM Cd in HEK293 cells expressing hTRPA1. At a holding potential of −60 mV, an inward current with an outwardly rectifying current–voltage relationship by ramp pulses from −100 mV to +80 mV every 5 s was observed (Figure 7D). Functional hTRPA1 expression was confirmed by the response to 50 μM ally isothiocyanate (AITC, a TRPA1 agonist). In two Zn-insensitive mutant channels (H983A, C641S/C1021S), TRPA1 activation by Cd (10 μM) was almost abolished although the responsiveness of AITC was intact (Figure 7B, C and E). These results suggested that the cysteine and histidine residues are important for the activation of TRPA1 by Cd, the manner of which is similar to Zn.

### Cd causes acute pain via activation of TRPA1

**Figure 8:**
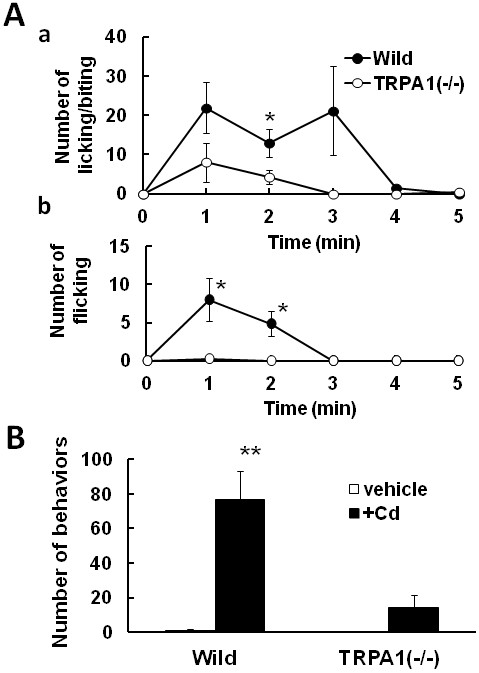
**Acute pain in mice induced by Cd. **(**A**) Changes in number of pain-related behaviors (a; licking and biting, b; flicking) of wild-type and TRPA1(−/−) mice after intraplantarly injection of Cd (2 nmol/paw). (**B**) Summarized number of behaviors during 5 min after Cd injection and HEPES-buffered solution, as a vehicle. Symbols with vertical lines show mean ± SEM. (wild-type; n=7, TRPA1(−/−); n=8) *,P <0.05.

We showed that Cd stimulated mouse sensory neurons through TRPA1 *in vitro*. Next, we examined whether Cd could actually induce acute pain *in vivo*. Intraplantar injection of Cd (2 nmol/paw) caused licking, biting (Figure 8Aa) and flicking (Figure 8Ab) of the injected paw as pain-related behaviors. These nociceptive behaviors began just after its application and ceased within 5 min. Figure 8B shows the total number of nociceptive behaviors for 5 min after the Cd administration in wild-type and TRPA1(−/−) mice. TRPA1(−/−) mouse displayed a significant attenuation of Cd-induced nociception. In a control experiment, no responses were observed in mice injected with the same amount of HEPES-buffered solution as a vehicle. These results indicated that activation of TRPA1 was associated with pain or irritation induced by Cd.

## Discussion

The present study indicates that Cd induced [Ca^2+^]_i_ increases in mouse primary sensory neurons, which were also sensitive to the TRPA1 agonist cinnamaldehyde and selectively inhibited by TRPA1 blockers. The Cd-induced [Ca^2+^]_i_ responses were absent in TRPA1 (−/−) mouse DRG neurons. [Ca^2+^]_i_ responses to Cd were confirmed in RIN-14B cells expressing TRPA1 endogenously. Cd evoked current responses in heterologously expressed TRPA1. In wild-type mice, intraplantar injection of Cd induced pain-related behaviors, which were largely attenuated in TRPA1 (−/−) mice. These results suggested that Cd elicited acute pain through the activation of TRPA1.

It is known that Cd and Zn bind cysteine and histidine residues in metallothionein, a cysteine-rich metal-binding protein [27], and in some metal ion transporters [28]. Zn directly activates heterologously expressed TRPA1 [21,22] and specific intracellular cysteine and histidine residues of TRPA1 bind Zn [22]. Our findings indicated that two cysteine (C641, C1021) residues and one histidine (H983) residue affected Cd sensitivity, since mutant TRPA1 channels (C641S/C1021S, H983A) showed attenuation of Cd sensitivity like Zn. These properties were confirmed by the patch-clamp experiments using heterologously expressed mutant TRPA1 channels. These amino acid residues were located in the intracellular domain and the N-terminal C641 has been identified as a residue involved in some reactive chemicals [29]. Thus, it seems likely that Cd activates TRPA1 through recognition of the same specific amino acid residues for its recognition sites as those for Zn.

Furthermore, Cd imaging using Leadmium Green showed that Cd entered mouse sensory neurons. The increases of fluorescent intensity of Leadmium Green in TRPA1(−/−) mouse DRG neurons were significantly lower than in wild-type ones. These results suggested that Cd may be able to permeate into neurons though TRPA1. It was confirmed in human TRPA1-expressing HEK293 cells. It has been reported that Zn permeates through TRPA1 and acts on the inner domain of this channel [22]. In TRPA1(−/−) mouse DRG neurons, however, Cd influx remained at higher concentrations, suggesting that Cd might enter through other pathways. It has been reported that Cd permeates through voltage-dependent Ca channels (L-type; [25], T-type; [30]), an Fe transporter (DMT1; [31]), Zn transporters (ZIP8; [32], ZIP14; [33]) and TRPV6 [34]. These channels or transporters are reported to be expressed in neurons [33,35-37]. Thus, in wild-type mouse DRG neurons, Cd entering through these pathways might also activate TRPA1.

In this study, we used 30–300 μM Cd, concentrations that were much higher than reported blood or urine Cd levels in exposed humans (blood-Cd:10 μg/L, urine-Cd:7 μg/L creatinine, [38]). It is reported that workers exposed to Cd fumes at 8.63 mg/m^3^ for 5 h exhibited symptoms of coughing and slight irritation of the throat and mucosa [39]. In the case of acute Cd exposure such as airway instillation, local concentrations would be higher than blood or urine concentrations. Similarly, in research on acute toxicity to Zn *in vitro*, submillimolar concentrations were used (300 μM; [40], 30 μM; [22], 30 μM, [23]). In the present study, intraplantar injection of Cd (2 nmol/paw), the amount of which was somewhat higher than reports for Zn in *in vivo* experiments (0.6 nmol, intraplantarly; [22], 0.05 nmol, intratracheally; [23]), elicited nociceptive behaviors in wild-type mice. On the other hand, Cd induced significantly fewer behavioral changes in TRPA1(−/−) mice, suggesting the involvement of TRPA1 in Cd-induced acute pain.

Recently, functional TRPA1 expression has been reported in non-neuronal cells such as lung fibroblast cells, epithelial cells and smooth muscle cells, which release IL-8 in response to TRPA1 agonists and contribute to lung inflammation [41]. It is also reported that Cd promotes secretion of IL-8 and IL-6 from airway epithelial cells [42]. For lung inflammation, therefore, not only neuronal but also non-neuronal TRPA1 may be involved in Cd toxicity.

It is reported that Cd produces reactive oxygen species (ROS) [43] that mediates Ca signaling involved in Cd-induced cell death [44]. Since ROS are also known to activate TRPA1 [13,45], we examined whether Cd elicited ROS production in mouse DRG neurons. However, Cd failed to produce ROS under our experimental conditions (30 or 300 μM, 2 min), using CM-H_2_DCFDA, a fluorescent ROS indicator (data not shown).

## Conclusions

The present study demonstrates that Cd excites sensory neurons via activation of TRPA1 and causes acute pain, the mechanism of which may be similar to that of Zn. Our present data show that TRPA1 contributes to the nociceptive or inflammatory effects of Cd. However, further studies are necessary to completely understand the pathological conditions of acute Cd toxicity.

## Methods

All protocols for experiments on animals were approved by the Committee on Animal Experimentation of Tottori University (♯11–T–2). All efforts were made to minimize the number of animals used.

### Isolation and culture of mouse DRG neurons

We used adult mice of either sex (4–16 weeks old). C57BL/6 J mice, TRPA1-null mice (kindly provided by Dr. D. Julius, University of California) were euthanized by inhalation of CO_2_ gas. Mouse DRG cells were isolated and cultured as described previously [46]. In brief, DRG cells were removed, dissected and freed from connective tissue under a dissecting microscope in phosphate-buffered saline (PBS: in mM, 137 NaCl, 10 Na_2_HPO_4_, 1.8 KH_2_PO_4_, 2.7 KCl) supplemented with 100 U/ml penicillin G and 100 μg/ml streptomycin. Then isolated ganglia were cut into small pieces and enzymatically digested for 30 min at 37°C in PBS containing collagenase (1 mg/ml, type II, Worthington, USA) and DNase I (1 mg/ml, Roche Molecular Biochemicals, USA). Subsequently, the ganglia were immersed in PBS-containing trypsin (10 mg/ml, Sigma, USA) and DNase I (1 mg/ml) for 15 min at 37°C. After enzyme digestion, the enzyme-containing solution was aspirated and the ganglia were washed with culture medium (Dulbecco’s-modified Eagle’s medium [DMEM, Sigma] supplemented with 10% fetal bovine serum [Sigma]), penicillin G (100 U/ml) and streptomycin (100 μg/ml). DRG cells were obtained by gentle trituration with a fine-polished Pasteur pipette. Then the cell suspension was centrifuged (800 rpm, 2 min, 4°C) and the pellet-containing cells were resuspended with the culture medium. Aliquots were placed on glass cover slips coated with poly-_D_-lysine (Sigma) and cultured in a humidified atmosphere of 95% air and 5% CO_2_ at 37°C. In the present experiment, cells cultured within 24 h were used.

### Culture of RIN-14B cells

The RIN-14B cells were purchased from DS Pharma Biomedical Co., Ltd. (Osaka, Japan). Cells were cultured in RPMI1640 medium (Wako) supplemented with 10% FBS, 100 U/ml penicillin G and 100 μg/ml streptomycin.

### Heterologous expression in HEK 293 cells

Cells were transfected using 1 μg of human TRPA1 (hTRPA1, gift from Ardem Patapoutian) and mutants of hTRPA1 (C641S/C1021S, H983A [47]), which were made using a modified QuickChange Site-Directed Mutagenesis method (Stratagene, La Jolla, CA, USA). Human embryonic kidney (HEK) 293 cells were cultured in DMEM supplemented with 10% FBS, 100 U/ml penicillin G and 100 μg/ml streptomycin. Cells were transfected with the expression vectors using a transfection reagent (Lipofectamine 2000 or Lipofectamine Reagent together with Plus Reagent, Invitrogen) and used 24 h after transfection.

### Calcium imaging

The intracellular Ca imaging in individual cells were performed with the fluorescent Ca indicator fura-2 by dual excitation using a fluorescent-imaging system controlling illumination and acquisition (Aqua Cosmos, Hamamatsu Photonics, Hamamatsu, Japan) as described previously [19]. Briefly, to load fura-2, cells were incubated for 40 min at 37°C with 10 μM fura-2 AM (Molecular Probes, Eugene, Oregon, USA) in HEPES-buffered solution (in mM: 134 NaCl, 6 KCl, 1.2 MgCl_2_, 2.5 CaCl_2_, and 10 HEPES, pH 7.4). A coverslip with fura-2-loaded cells was placed in an experimental chamber mounted on the stage of an inverted microscope (Olympus IX71) equipped with an image acquisition and analysis system. Cells were illuminated every 5 s with lights at 340 and 380 nm, and the respective fluorescence signals of 500 nm were detected. The fluorescence emitted was projected onto a charge-coupled device camera (ORCA-ER, Hamamatsu Photonics) and the ratios of fluorescent signals (F340/F380) for [Ca^2+^]_i_ were stored on the hard disk of a computer (Endeavor pro 2500, Epson). Cells were continuously superfused with HEPES-buffered solution at a flow rate of ~2 ml/min through a Y-tube pipette. The composition of high-K solution was (in mM) 80 KCl, 1.2 MgCl_2_, 2.5 CaCl_2_, and 10 HEPES, pH 7.4). For Ca-free external solution, Ca was omitted. All experiments were carried out at room temperature (22-25°C).

### Whole-cell current recording

HEK293 cells expressing hTRPA1 and mutants of hTRPA1 on coverslip were mounted in an experimental chamber and superfused with HEPES-buffered solution as for Ca imaging experiments. The pipette solution contained (in mM: 140 KCl, 1.2 MgCl_2_, 2 ATPNa_2_, 0.2 GTPNa_3_, 10 HEPES, 10 EGTA, pH 7.2 with KOH). The resistance of patch electrodes ranged from 4 to 5 MΩ. The whole-cell currents were sampled at 5 kHz and filtered at 1 kHz using a patch-clamp amplifier (Axopatch 200B; Molecular Devices, Sunnyvale, CA) in conjunction with an A/D converter (Digidata 1322A; Molecular Devices). Membrane potential was clamped at −60 mV and voltage ramp pulses from −100 mV to +80 mV for 100 ms were applied every 5 s.

### Cadmium imaging

For single-cell Cd imaging, we used Leadmium Green (Molecular Probes), a specific indicator for lead and cadmium. To load Leadmium Green, cells were incubated for 40 min at 37°C with 50 ng/μl Leadmium Green-AM in HEPES-buffered solution. Cd imaging was performed using the same apparatus as for Ca imaging. Cells were illuminated with light at 490 nm and fluorescence signals of 520 nm were collected. For Cd imaging, cells were superfused with Ca-free external solution. Signals were expressed as the relative change in fluorescence, F/F_0_, where F and F_0_ indicate fluorescence at any given period and the initial period, respectively.

### Behavioral experiment

Mice were placed in cages for 30 min before experiments. Twenty microliters of the HEPES-buffered solution (vehicle), which was similar in composition to that used in *in vitro* experiments, was first injected intraplantarly into the right hind paw as a control. The numbers of times each mouse licked, bit and flicked the injected paw were counted for 15 min after the injection. Subsequently, the same amount of Cd (2 nmol/paw) was injected into the left hind paw and the number of pain-related behaviors were counted for 15 min.

### Chemicals

The following drugs were used (vehicle and concentration for stock solution). Capsaicin (ethanol, 0.01 M), HC-030031 (dimethyl sulfoxide: DMSO, 0.01 M), AP18 (DMSO, 0.1 M) and Cremophor EL (distilled water: DW, 1%) were from Sigma. Ruthenium red (DW, 0.01 M), cadmium chloride (DW, 1 M) and zinc chloride (DMSO, 0.1 M) were from Wako (Osaka, Japan). N-(4-t-butylphenyl)-4-(3-chloropyridin-2-yl)tetrahydropyrazine-1(2H)-carboxamide (BCTC, DMSO, 0.01 M) was from BIOMOL Research Laboratories, Inc., (Pennsylvania, USA). A967079 (DMSO, 0.01 M) was from Focus Biomolecules, Llc (Pennsylvania, USA). N,N,N',N'-tetrakis-(2-pyridylmethyl) ethylenediamine (TPEN, 0.05 M) was from Dojindo Molecular Technologies, Inc.,(Kumamoto, Japan). Cinnamaldehyde (DMSO, 1 M) was from Nacalai Tesque, Inc. (Kyoto, Japan). All other drugs used were of analytical grade.

### Data analysis

The data are presented as the mean ± SEM (n=number of cells). For comparison of two groups, data were analyzed by the unpaired Student’s *t* test, and for multiple comparisons, one-way ANOVA following by Turkey-Kramer test was used. Differences with a *P*-value of less than 0.05 were considered significant. Values of the 50% effective concentrations (EC_50_) were determined using Origin version 9.0 J (Origin-Lab).

## Abbreviations

Cap: Capsaicin; DRG: Dorsal root ganglion; DMSO: Dimethyl sulfoxide; [Ca2+]i: Intracellular Ca^2+^ concentration; TRPA1: Transient receptor potential ankyrin 1; TRPV1: Transient receptor potential vanilloid 1.

## Competing interests

The authors declare that there are no conflicts of interest.

## Authors’ contributions

SM carried out all of the experiments and majority data analysis. KT participated in some of the data analysis. TI and KU prepared experimental materials. SS, MT, TO and SM conceptualized the project and formulated the hypothesis and wrote the manuscript. TO designed and directed the experiments. All authors read and approved the final manuscript.
